# Intramolecular Photo-Oxidation as a Potential Source to Probe Biological Electron Damage: A Carboxylated Adenosine Analogue as Case Study

**DOI:** 10.3390/molecules26102877

**Published:** 2021-05-13

**Authors:** Maria Elena Castellani, Jan R. R. Verlet

**Affiliations:** Department of Chemistry, Durham University, Durham DH1 3LE, UK; maria.e.castellani@durham.ac.uk

**Keywords:** photoelectron spectroscopy, low energy electron damage in DNA, charge transfer

## Abstract

A carboxylated adenosine analog (C-Ado^−^) has been synthesized and probed via time-resolved photoelectron spectroscopy in order to induce intra-molecular charge transfer from the carboxylic acid moiety to the nucleobase. Intra-molecular charge transfer can be exploited as starting point to probe low-energy electron (LEE) damage in DNA and its derivatives. Time-dependent density functional theory (TD-DFT) calculations at the B3LYP-6311G level of theory have been performed to verify that the highest occupied molecular orbital (HOMO) was located on carboxylic acid and that the lowest occupied molecular orbital (LUMO) was on the nucleobase. Hence, the carboxylic acid could work as electron source, whilst the nucleobase could serve the purpose of electron acceptor. The dynamics following excitation at 4.66 eV (266 nm) were probed using time-resolved photoelectron spectroscopy using probes at 1.55 eV (800 nm) and 3.10 eV (400 nm). The data show rapid decay of the excited state population and, based on the similarity of the overall dynamics to deoxy-adenosine monophosphate (dAMP^–^), it appears that the dominant decay mechanism is internal conversion following ^1^ππ* excitation of the nucleobase, rather than charge-transfer from the carboxylic acid to the nucleobase.

## 1. Introduction

DNA is one of the most important biomolecules, as it contains genetic information that is essential to most forms of life. DNA can suffer damage from different sources, for example radiation or oxidizing agents [1,2,3]. For high-energy radiation such as X-rays used in radiography or beta-radiation emitted in the Chernobyl disaster 35 years ago, it is the secondary products rather than the primary radiation that causes the largest disruptions [4,5]. The high-energy radiation ionizes predominantly water to form secondary electrons with a kinetic energy (eKE) between 0 and 20 eV. These so-called low energy electrons (LEEs) are responsible for severe DNA lesions, such as single- and double-strand breakages, which can ultimately lead to cell death and disease [6].

LEEs can attach to any of the nucleotide’s components, i.e., the nucleobase, sugar and phosphate [7,8,9]. At very low energy, LEEs can attach as a dipole bound state (DBS) outside the molecular framework [10,11]. LEE attachment can lead to the formation of metastable anionic states known as temporary negative ions or resonances, which are responsible for inducing mutagenesis in living organisms [12,13]. Boudaiffa et al. [14] showed that single-strand breaks are caused by core-excited (Feshbach) resonances, which are produced by electron attachment to the π* orbitals of the nucleobases. Conversely, electrons with eKE < 3 eV cause the formation of shape resonances [7,15,16], in which dissociative channels, involving mainly dehydrogenation, but also cleavage of the phosphodiester and glycosidic bonds, are active [4,6,17,18,19]. The mechanism comprises electron migration from the π* orbital on the nucleobase to the σ* orbital of either the C-O, N_1_-C or N-H bond, with subsequent dissociation of the σ bond [4,20,21,22]. Computational and experimental evidence [23] suggests that electrons with energy > 2 eV can occupy the π* orbital of the phosphate group and contribute to single-bond breaks. Moreover, due to its low energy barrier and the high electron affinity (EA) of the phosphate group, the phosphodiester bond, together with the N_1_-C bond, is the most probable to undergo cleavage caused by shape resonances [24].

LEE-induced damage has been extensively studied by Sanche and co-workers through electron scattering measurements, in which whole DNA samples are irradiated with an electron beam, and then the products, formed across a spectrum of incoming electron energies, are probed [6,9,12,13,14,21,22,23,24,25]. However, in order to probe the dynamics of the processes and thus offer a direct experimental window into the mechanisms at play, time-resolved spectroscopy is required. Probing LEE-driven chemistry in the time-domain remains challenging. One approach is to access the resonances from an anionic ground state and subsequently probe the dynamics using time-resolved photoelectron spectroscopy [26,27]. We have developed and exploited such methods—however, they are limited to molecules that have a positive electron affinity. An alternative method involves the use of iodide as a source of LEEs. Specifically, the Johnson group showed how photoexcitation of iodide-molecule clusters accesses the dipole-bound state of the clustered molecule [28,29,30,31,32,33]. The Neumark group [34,35,36,37,38] has elegantly extended this method to the time-domain using time-resolved photoelectron imaging, including on I^−^•N (N = nucleobase) clusters. However, these methods are limited by two main factors: (1) the iodine remains present and may be more than a mere spectator and (2) the excitation energy of the charge-transfer transition from iodide is at relatively high photon energies that often coincide with absorption bands on the nucleobase. Still, this is an excellent approach to probing the role of non-valence states in electron capture [39,40].

In the present study, we explore the feasibility of a new approach based on the use of an intra-molecular electron source using photo-induced charge-transfer. In principle, a nucleotide would be an ideal candidate, given that the phosphate is negatively charged: one might envisage the possibility of performing a charge-transfer transition to the nucleobase, then subsequently probing the dynamics using time-resolved photoelectron spectroscopy. However, the electron affinity of the phosphate group is very large and would require *hv* > 5 eV [41,42]. Instead, here we explore the use of a different source of electrons based on a carboxylic acid. Specifically, following the method by Widlanski and Epp [43], we synthesized an adenosine analog with a carboxylic group in 5′ position (C-Ado). The –OH moieties of the sugar in position 2′ and 3′ are protected with an isopropyl function, in order to prevent deprotonation. If the highest occupied molecular orbital (HOMO) is located on the carboxylic acid, one could envision a charge-transfer transition occurring from the carboxylic acid moiety to the π* state on the nucleobase.

## 2. Results

**Figure 1:**
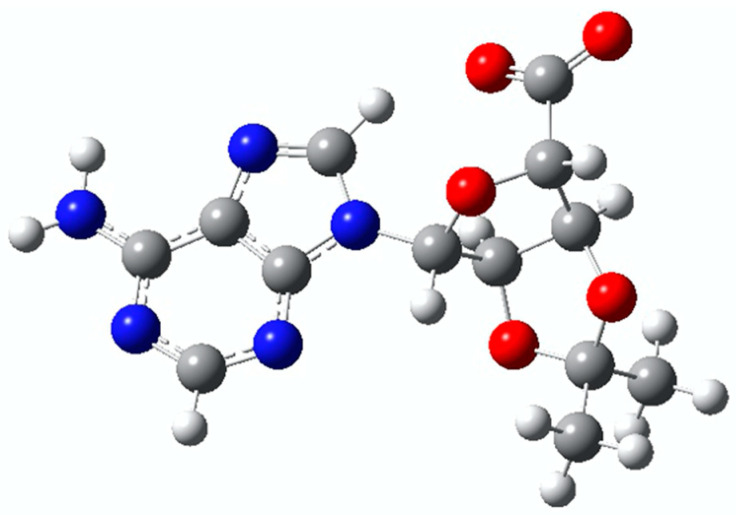
Minimum energy structure of C-Ado^−^ calculated at the B3LYP-6311G level of theory. The hydrogen atom is white, carbon is grey, nitrogen is blue, and oxygen is red.

To assess whether the electronic structure of C-Ado is suitable, density function theory (DFT) calculations were performed. The minimum energy structure of C-Ado^−^ was obtained at the B3LYP-6311G level of theory and is shown in Figure 1. As the hydroxyl groups on the ribose are inaccessible due to the protective group, the only deprotonation site is the carboxyl group, and this is where the negative charge is hosted. The calculated vertical detachment energy (VDE) and adiabatic detachment energy (ADE) resulted, respectively, 4.48 eV and 4.29 eV.

**Figure 2:**
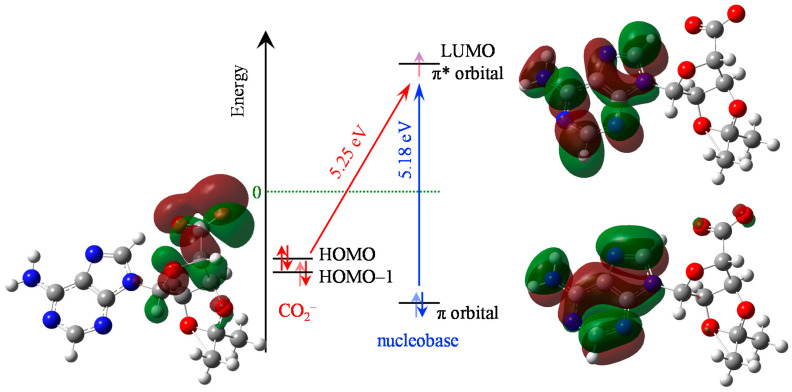
Energy level diagram and transitions for C-Ado^−^, showing the carboxy-localized molecular orbitals (red/green transparent shading, HOMO and HOMO–1) as well as the nucleobase-localized molecular orbitals (π and π* MOs). The two relevant transitions are shown with the charge-transfer (red diagonal arrow) and the nucleobase-centered ππ* (blue vertical arrow). Atoms have same colours as in Figure 1.

Time-dependent DFT calculations were used to compute the molecular orbitals (MOs). The highest occupied MO (HOMO), lowest unoccupied MO (LUMO), and relevant π MOs of C-Ado^−^ are shown in Figure 2 along with the relevant energetics. The HOMO, as expected, is predominantly located on the carboxylic acid moiety, with some charge density extending onto the ribose sugar. The initial state for the charge-transfer transition and is also hosted by the carboxyl group and is the HOMO-1 but it is essentially degenerate to the HOMO. The LUMO is an anti-bonding π* orbital of the nucleobase, as can be seen in Figure 2. This is the lowest resonance of the nucleobase (shape resonance). The lowest energy charge-transfer transition from the carboxylic acid to the nucleobase excites this π* orbital and so, in principle, such an excitation can directly mimic electron capture via the shape resonance. However, the π* orbital is also involved in the ππ* transition localized on the nucleobase. The ππ* transition is calculated at 5.18 eV (239 nm), while the charge transfer transition is higher, at 5.25 eV (236 nm). Hence, both transitions are very close in energy according to the calculations. We stress however that the calculations are not intended to offer quantitative insight and rather to obtain some qualitative information on the relevant transitions and their relative energies.

**Figure 3:**
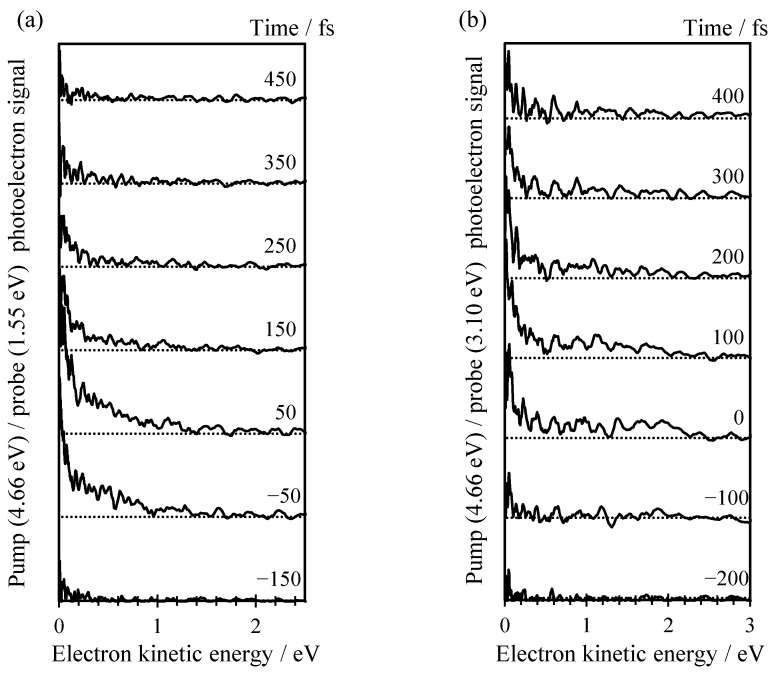
(**a**) Time-resolved photoelectron spectrum of C-Ado^−^ recorded with pump energy of 266 nm and probe energy of 800 nm between −150 and 450 fs; (**b**) Time-resolved photoelectron spectrum of C-Ado^−^ recorded with pump energy of 266 nm and probe energy of 400 nm between −200 and 400 fs. The maximum overlap between pump and probe pulse is taken as 0 fs reference.

The excited state dynamics following excitation of C-Ado^−^ at 4.66 eV (266 nm) were probed using time-resolved photoelectron imaging using both a 1.55 eV (800 nm) probe or a 3.10 eV (400 nm) probe. The results from these experiments are shown in Figure 3a,b, respectively. In Figure 3, the photoelectron signal from the pump only (i.e., before *t* = 0) has been subtracted from each time-resolved spectrum to leave only the temporally evolving dynamics.

In Figure 3a, two distinct photoelectron features can be observed at different kinetic energies. At very low KE (<0.15 eV), a narrow peak is seen near *t* = 0, which appears to have a shoulder extending out to ~1 eV. The dynamics of the higher KE feature appear to be faster than the low KE peak. In an attempt to probe a slightly larger Franck–Condon window, we also probed the same dynamics with a 3.10 eV probe as shown in Figure 3b. Unfortunately, these data are of slightly poorer signal-to-noise compared to the spectra with the 1.55 eV probe.

The time-resolved spectra in Figure 3b can be roughly divided into a number of distinct features. The first at low KE is similar to that observed with the 1.55 eV probe. It is a narrow peak with KE < 0.2 eV. There is also a feature between 0.2 < KE < 1.0 eV that seems to decay at a similar timescale to the low KE peak. At higher energy still, 1.0 < KE < 2.5 eV, a third feature can be discerned, but only near *t* = 0.

The time-resolved photoelectron spectrum at *t*~0 can be used to extract an approximate value of the ADE for C-Ado^−^. To improve the signal to noise, we have taken the average of three spectra at *t*~0 fs and from the maximum KE of these, we conclude that the ADE~5.2 eV for both the experiment with a 1.55 eV and the one with a 3.10 eV probe pulse. Note that the ADE determined in this manner is that associated with the initial excitation because it is determined using a resonance-enhanced scheme.

**Figure 4:**
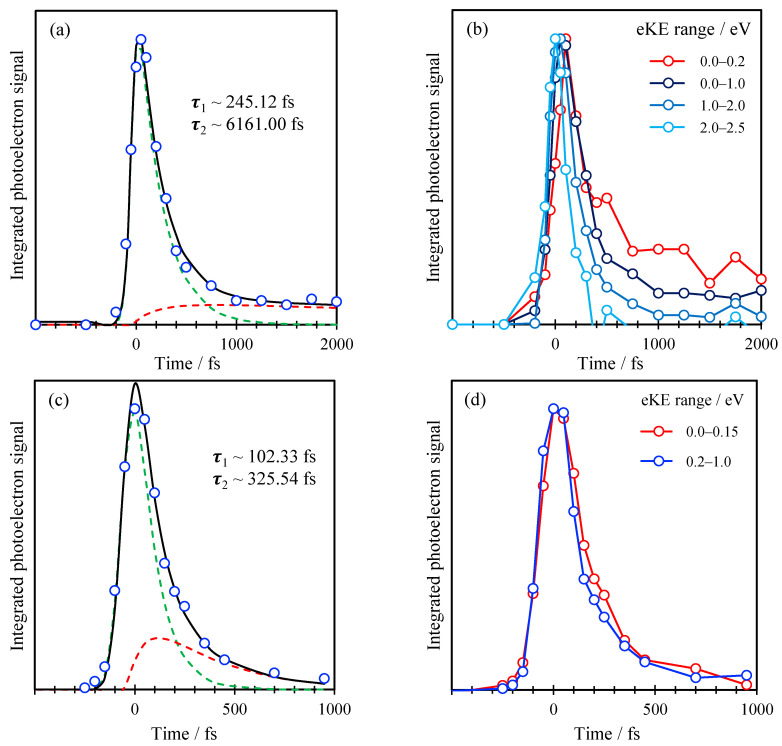
(**a**) Total integrated photoelectron signal with pump energy of 266 nm and probe energy of 400 nm (blue dots) plotted in function of time, with corresponding fits; (**b**) Integrated photoelectron signal with pump energy of 266 nm and probe energy of 800 nm over different energy ranges. (**c**) Same as (**a**) with pump energy of 266 nm and probe energy of 800 nm; (**d**) Same as (**b**) with pump energy of 266 nm and probe energy of 400 nm. Black lines in (**a**,**c**) are fit to total signal; dashed green and red lines are fits to kinetic model for the fast and slow component, respectively.

To analyze the decay dynamics of the spectral features identified in Figure 3, we consider the integrated photoelectron signal over specific spectral ranges. These are shown in Figure 4 for the data taken with a 1.55 eV probe in (a) and a 3.10 eV (b). In Figure 4a, the total integrated signal is shown along with the dynamics associated with the two features—the peak at low KE and the shoulder at 0.2 < KE < 1.0 eV. The latter have been normalized to a common maximum integrated intensity to allow for easy comparison of the dynamics of the individual features. Both features decay very rapidly and with essentially identical dynamics, suggesting that both peaks are a measure of the same excited state population dynamics. The total integrated dynamics are best fit to a sequential decay process, A→B→C, where both A and B are observable, and C is some final state that is not observable with the 1.55 eV probe pulse. The fit is included in Figure 4a and shows that the two decay lifetimes are *τ*_AB_ = 100 fs and *τ*_BC_ = 330 fs. Given that the instrument response function (cross-correlation of pump and probe) is ~100 fs, *τ*_AB_ is effectively within our time-resolution.

Performing a similar analysis for the data using a 3.10 eV probe is shown in Figure 4b. The total integrated photoelectron signal can similarly be fitted to a sequential decay as shown, which yields decay lifetimes of *τ*_AB_ = 250 fs and *τ*_BC_ ~ 6.2 ps (although the latter has a very large error as we have not measured the complete decay of this signal). However, dividing up the spectral ranges into representative slices, as shown in Figure 4b, reveals that the decay lifetime scales inversely with the KE of the photoelectron signal. Specifically, for 2.0 < KE < 2.5 eV, the signal decays very fast and essentially appears as the Gaussian instrument response function. The decay for 1.0 < KE < 2.0 eV is slightly slower with a small offset at longer times which becomes even slower for 0.2 < KE < 1.0 eV and with a larger long-time offset and again for the 0.0 < KE < 0.2 eV range. Additionally, it is clear that the rise of the signal is delayed at lower KE, suggesting that the photoelectron signal is effectively shifting to lower KE as time progresses. Hence, even though the fit to the sequential decay is good, it does not capture the true dynamics taking place.

## 3. Discussion

In line with our design aims, calculations show that the HOMO of C-Ado^−^ is predominantly localized on the carboxylic acid and the LUMO/π* orbital on the nucleobase. Moreover, a charge-transfer transition calculated to lie at ~5.25 eV is accessible and can, in principle, be used to drive an intramolecular charge injection onto the nucleobase. However, it is also clear that the ππ* transition on the nucleobase alone is also near to this computed energy and would provide an alternative to the intended charge-transfer. Nevertheless, our computations were used predominantly as a guide of the electronic structure rather than an absolute indicator and we note that much higher-level calculations have been used in literature [41]. In particular, calculations of transition energies may be expected to have large associated errors. Perhaps surprising is the poor agreement between the computed ADE (4.29 eV) and that measured at ~5.2 eV. However, it is important to underline that the experimental value does not necessarily correspond to the computed ADE. As the HOMO is localized predominantly on the carboxylic acid, the computed ADE corresponds to the removal of an electron from this site. The experimental ADE is derived from a resonance-enhanced measurement. If the initial excitation is localized on the nucleobase, then the measured ADE will correspond to the ionization energy of the nucleobase in C-Ado^−^. Note that the measured ~5.2 eV is not that dissimilar to the ionization of adenine in the phosphorylated nucleotide, dAMP^−^, measured to be 5.65 ± 0.15 eV [41,42]. Hence, it is important to know where the initial excitation takes place, for which the time-resolved measurements may provide some insight.

The time-resolved measurements in Figure 4 show that the dynamics are rapid and that the 1.55 eV probe is probing only a small fraction of the excited state evolution. We therefore focus the discussion on the dynamics observed with the 3.10 eV probe. This shows that the initially excited is very short lived and appears to shift towards lower KE on a timescale of a few 100s fs. Overall, these dynamics are similar to those observed for dAMP^−^ [41,42] following excitation and probing at the same energies. While it is difficult to correlate the exact timescales, the overall shifting towards lower KE also occurs in dAMP^−^ and this was assigned to motion on the ππ* state away from the Franck–Condon region. That population then decays on a 290 fs timescale by internal conversion leaving no excited state photoelectron signal beyond ~1 ps. The same population dynamics are also seen in bare adenine, 9-methyl-adenine, adenine oligonucleotides and adenine triphosphate dianions [42,44,45,46,47]. Hence, it is not wholly unsurprising that similar overall dynamics are observed in C-Ado^−^.

While the initial dynamics are similar, it is apparent from Figure 4b that some population remains after 1 ps. It should be noted that this signal is at very low KE, where noise is most severe and, therefore, there is some uncertainty in this signal and certainly in its subsequent decay. Nevertheless, it does appear that there are differences between the observed dynamics in dAMP^−^ and C-Ado^−^ at longer times. The main difference between the two systems is the availability of the charge-transfer transition that is predicted at a very similar excitation energy as the ππ* state in C-Ado^−^. Hence, the low KE signal seen in C-Ado^−^ for *t* > 1 ps could, in principle, be associated with population arising from charge-transfer. The excited state produced following such an excitation would leave the negative charge localized on the nucleobase and appears to have a longer lifetime. However, our data are of insufficient quality to analyze the evolution nor to be certain that the charge-transfer transition is excited.

Taking the above observations together, it appears that the majority of the excitation energy excites population into the nucleobase-centered ππ* state. This then decays in a similar manner as other adenine and purine derivatives [44,45,48]. The lack of very clear evidence for differing dynamics may be due to the oscillator strength for direct charge-transfer excitation from the carboxylate to the nucleobase being much smaller than that for the ππ* transition. Hence, despite lowering the electron affinity of the compound, substitution of the phosphate group with a carboxylic acid does not red-shift the charge-transfer transition sufficiently to avoid the ππ* transition. Nevertheless, we believe that the principle remains valid, and that the present study offers valuable lessons moving forward in the design of nucleotide-derivatives with different substituents. Specifically, one should aim to reduce the EA further for the anion group and to reduce the transition energy of the charge-transfer state. Possible modification could include the incorporation of hydroxyl groups that have been observed to lower the EA in substituted benzene derivatives [49]. Alternatively, on might consider attaching a nearby chromophore whose excited state has a similar energy to the π* MO on the nucleobase to affect charge-transfer. The current preliminary study is intended to serve as a stepping-stone for future work, which will aim to understand how to exploit intra-molecular charge transfer as a probe for low-energy electron damage in DNA and its derivatives, but it may open up exciting opportunities to probe electron-driven chemistry in DNA. Coupling this experimental approach with quantum dynamics calculations, particularly surface hopping, could help in better understanding the extent of charge-transfer in DNA. [50,51,52,53]

## 4. Materials and Methods

A detailed description of the instrument can be found in previous publications [54,55]. Carboxylated-5′-adenosine (C-Ado) was prepared following the protocol reported by Epp and Widlanski [43]. 1 mM of C-Ado was dissolved in methanol, adding few drops of NH_4_ in MeOH to facilitate deprotonation. The solution was pushed through a syringe into the first vacuum region of the apparatus, in which the anions were produced by electrospray ionization (ESI). Sets of ring-electrode ion guides guided the ions through a potential gradient towards a pulsed ion trap, then focused collinearly into a Wiley–McLaren time-of-flight mass spectrometer [56]. The mass-selected ion packet was irradiated with laser pulses obtained from a commercial Ti:Sapphire laser. The fundamental at 1.55 eV and the second harmonic at 3.10 eV were used as probe pulses for different experiments. The third harmonic was instead used as a pump pulse, with ~100 fs temporal resolution. The thus-produced photoelectrons were collected and imaged using a perpendicular velocity-map imaging (VMI) arrangement [57]. Onion peeling in polar coordinates was exploited for spectral extraction [58]. The well-known PE image of I^−^ was chosen as calibrant. Spectral resolution resulted in ∼5% of the kinetic energy.

Platinum plated VMI electrodes were used in order to ameliorate the signal-to-noise ratio, thanks to the higher work-function of platinum compared to stainless steel [59]. For background subtraction, as well as to increase PE signal, the PE images were collected with both the ion trap open and closed at ~1 Hz. A more detailed description of this procedure can be found here [46].

Time-resolved data were analyzed first by integration, then fitted to a given function to extract quantitative information. The model we use exploits exponentially modified Gaussian functions convoluted with the instrument response over time, which is given by the cross-correlation of the pump-probe signal. Details on the analytical expression of the fit can be found here [60].

Density functional theory (DFT) calculations have been performed with the software Gaussian09 [61], and the results visualized with GaussView 4. B3LYP (Becke three-parameter hybrid functional combined with Lee-Yang-Parr correlation functional) exchange correlation functional was used [41] together with the 6-311G basis set [62,63]. Diffuse and polarization functions were added. For molecular orbital visualization, the corrected functional CAM-B3LYP was instead used [64].

## Data Availability

Data is available from the authors upon reasonable request.
